# Evidence of recurrent selection of mutations commonly found in SARS-CoV-2 variants of concern in viruses infecting immunocompromised patients

**DOI:** 10.3389/fmicb.2022.946549

**Published:** 2022-07-26

**Authors:** Livia R. Goes, Juliana D. Siqueira, Marianne M. Garrido, Brunna M. Alves, Claudia Cicala, James Arthos, João P. B. Viola, Marcelo A. Soares

**Affiliations:** ^1^Oncovirology Program, Brazilian National Cancer Institute, Rio de Janeiro, Brazil; ^2^Laboratory of Immunoregulation, National Institute of Allergy and Infectious Diseases, Bethesda, MD, United States; ^3^Hospital Infection Control Committee, Brazilian National Cancer Institute, Rio de Janeiro, Brazil; ^4^Immunology and Tumor Biology Program, Brazilian National Cancer Institute, Rio de Janeiro, Brazil; ^5^Department of Genetics, Federal University of Rio de Janeiro, Rio de Janeiro, Brazil

**Keywords:** SARS-CoV2 infection, immunocompromised patient, VOC, long term virus shedding, spike, Nsp6, deletions/mutations

## Abstract

Chronically immunosuppressed patients infected with SARS-CoV-2 often experience prolonged virus shedding, and may pave the way to the emergence of mutations that render viral variants of concern (VOC) able to escape immune responses induced by natural infection or by vaccination. We report herein a SARS-CoV-2^+^ cancer patient from the beginning of the COVID-19 pandemic whose virus quasispecies across multiple timepoints carried several immune escape mutations found in more contemporary VOC, such as alpha, delta and omicron, that appeared to be selected for during infection. We hypothesize that immunosuppressed patients may represent the source of VOC seen throughout the COVID-19 pandemics.

## Introduction

Recent reports have shown that prolonged SARS-CoV-2 infections can lead to the highly mutated viruses due to continuous virus replication in the host (Avanzato et al., 2020; Choi et al., 2020; Kemp et al., 2021). Long virus shedding, defined by rt-PCR-positive tests for the presence of the virus for over 40 days of infection, has been particularly reported among immunocompromised patients, and likely result from the inability of an impaired immune system to control virus replication. In this context, immunocompromised hosts are thought to play an important role in the genesis and emergence of SARS-CoV-2 variants in human populations. In this report, we provide evidence for such a hypothesis, with the description of an immunocompromised patient from early in the COVID-19 epidemics that showed multiple viral mutations typical of contemporary VOC.

## Materials and methods

**FIGURE 1:**
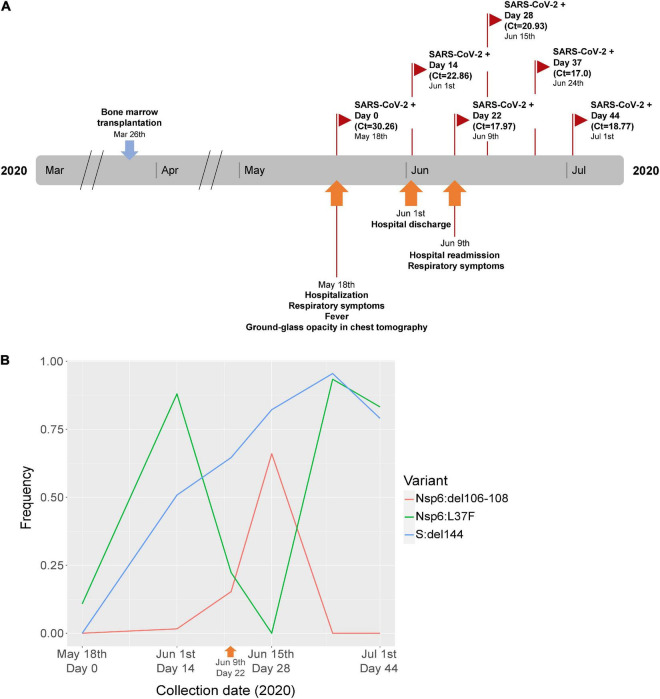
**(A)** Timeline of clinical events and sample collection in a cancer patient from the first wave of the COVID-19 pandemic in Brazil (2020). **(B)** Frequency of SARS-CoV-2 immune escape mutations at selected timepoints of the patient. In each timepoint of the graph, the relative frequency in the within-host quasispecies is depicted for all three mutations that appeared during the evolution of the virus across the infection of the patient (see legend at right for each mutation). Timepoints were collected at days 0, 14, 22, 28, 37, and 44 after COVID-19 diagnosis, and the sample sequenced (from day 22) is depicted with an orange arrowhead in the graph.

A 31-year old male patient with myeloid leukemia was admitted to the Brazilian National Cancer Institute at day 53 after a bone-marrow transplantation with respiratory symptoms, fever and a chest tomography showing a ground-glass profile. The patient tested positive in a SARS-CoV-2 RT-PCR test (Ct = 30.36) and was discharged after 14 days, still positive (Ct = 22.86). Twenty-two days after the first positive test, he was readmitted with new respiratory symptoms, still positive for the virus (Ct = 17.97). During the second hospitalization, the patient had three other SARS-CoV-2 positive tests at days 28 (Ct = 20.93), 37 (Ct = 17), and 44 (Ct = 18.77) after the first test (Figure 1A).

All positive samples were subjected to viral nucleic acid isolation, and PCR amplification and sequencing of the full-length genome were performed as previously described (Siqueira et al., 2021). Generated reads were analyzed using Geneious R11 (Biomatters, Auckland, New Zealand). First, bases with quality below 30 Phred scores and reads shorter than 60 bp were trimmed out using BBduk plugin. Reads were then assembled to the Wuhan-Hu-1 reference sequence genome (GenBank #MN908947). Consensus sequences were extracted and the different timepoints were compared. Single nucleotide variant (SNV) positions identified between the longitudinal consensuses were evaluated for minor nucleotide frequency using LoFreq v.2.1.5 (Wilm et al., 2012). Variants supported by a minimum of 75x depth coverage and frequency of 2% were considered as non-spurious according to Popa et al. (2020).

Single genome amplification (SGA) was performed with the sample collected on June 9th (day 22) (Figure 1B) to confirm the results found by full-length genome next generation sequencing. The SGA protocol was adapted from Kijak et al. (2019) using nested PCR with primers 35L/39R (first round) and 36L/38R (second round) for nsp6 amplification and primers 72L/74R (first round) and 72L/73R (second round) to amplify a fragment of spike gene. Primers were selected from the ARTIC network n-Cov-2019 V.3 primer set.^1^ All PCR reactions were carried out with Platinum Taq DNA polymerase high fidelity following manufacturer instructions and 2 μl of diluted cDNA or first round PCR product. The cycling conditions were 94°C for 5 min, followed by 40 cycles of 94°C for 15 s, 60°C for 30 s and 68°C for 105 s, and a final extension of 72°C for 5 min. Sequences were generated by Sanger sequencing using an automated ABI 3130xl Genetic Analyzer (Thermo Fisher Scientific, Waltham, MA, Unites States) and assembled to Wuhan-Hu-1 SARS-CoV-2 (Genbank acc# MN908947) using Lasergene package (DNAStar, Inc., Madison, WI).

## Results

All six timepoints of the patient were sequenced and the mean coverage depth varied between 1,442.2 and 2,132.1x across the SARS-CoV-2 near full-length genome. The timepoint sequences showed a fluctuation of one SNV and two in-frame deletions over time (Figure 1B). The L37F SNV located in Nsp6 was found at a frequency of 0.10 at the first time point. Fourteen days after diagnosis, this SNV was observed at a frequency of 0.88, with a decrease at days 22 and 28, followed by an increase at day 37 (0.93) and maintenance at day 44 (0.83). A three base-pair deletion corresponding to the amino acid residue 144 of the Spike protein (originally encoding a tyrosine in the Wuhan-Hu-1 prototype sequence) (S:del144) was first detected at the second timepoint (day 14 after diagnosis) at a frequency of 0.58 and showed increased frequency over time. A nine base-pair deletion in NSP6 encoding three amino acid residues (originally a serine-glycine-phenylalanine in the Wuhan-Hu-1 prototype sequence) located within ORF1 (Nsp6:del106-108) was first observed at the second timepoint with a 0.016 frequency, showing an increased frequency at day 28 (0.66), but no detection by days 37 and 44 after diagnosis. SGA sequencing from the sample collected at day 22 confirmed the findings for this sample, where Nsp6:L37F and Nsp6:del106-108 were found at minor frequency (25 and 37.5%, respectively) among the eight nsp6 amplicons obtained from 1:13,122 and 1:19,683 dilutions, respectively. For S:del144, six amplicons were sequenced from a 1:6,561 dilution and four showed the presence of the spike deletion. The remaining two showed an eletropherogram profile suggestive of the presence of both deleted and non-deleted spike sequences (multiples peaks suggesting overlapping of different sequences 3′ to the position of the deletion in the forward primer and 5′ to the deletion in the reverse primer). These results corroborate the NGS observations, showing that most sequences from spike have carry the S:del144 mutation.

## Discussion

In this report, we provide evidence that SARS-CoV-2 infecting an immunocompromised host (in our case, a cancer patient) generates several mutations during prolonged infection that are characteristic of VOC, even before any known VOC had been documented. The S:144del mutation is a signature of the Alpha and Omicron VOC (Viana et al., 2022). It is located in the N-terminal domain antigenic-supersite of the Spike protein and is associated with viral immune evasion (Cerutti et al., 2021). The L37F mutation of Nsp6 has been associated with reduced activation of inflammasomes and of pyroptosis (Sun et al., 2022), and has also been associated with asymptomatic SARS-CoV-2 infections (Wang et al., 2020). The Nsp6:del106-108 is found in multiple VOC (Alpha, Beta and Gamma), but its impact is unknown. Nsp6 has also been reported as associated with viral immune evasion (Xia et al., 2020).

The slight differences found in the frequencies of mutations when analyzed by NGS and SGA for the two in-frame deletions (Nsp6:del106-108 and S:del144) could be explained by the low number of amplicons sequenced in the SGA methodology or, alternatively, a bias of the NGS assembling, where a non-deleted sequence (Wuhan-Hu-1 SARS-CoV-2) was used as reference.

Because immunosuppressed patients, such as cancer or HIV patients, are more prone to have prolonged SARS-CoV-2 shedding (Avanzato et al., 2020) due to ongoing virus replication, they are putatively central to the genesis of SARS-CoV-2 variants, and perhaps VOC. Indeed, it has been suggested that VOC carrying many novel mutations appearing at once, as were the cases of Alpha and Omicron, were generated within chronically infected subjects with immune responses impaired by other underlying illnesses (Kupferschimidt, 2021). Similar to our work, a South African woman with an uncontrolled HIV infection was reported as infected with a SARS-CoV-2 carrying several mutations commonly seen in VOC (Karim et al., 2021). Most strikingly, however, is that the cancer patient herein described was diagnosed with a virus carrying VOC mutations during the first wave of the pandemic in Brazil (between May and July 2020), suggesting that COVID-19 patients with underlying chronic conditions are able to generate SARS-CoV-2 with immune evasion mutations throughout the entire pandemic. Consistent with this notion is the fact that Omicron variant, despite being the latest recognized VOC, had its most recent ancestor estimated to early 2020, at the beginning of the COVID-19 pandemic.

The observation that immunosuppressed carriers are pivotal to the genesis of viral mutations that can be selected for and lead to increased virus fitness and immune escape is not exclusive to SARS-CoV-2, but has been described for other RNA viruses. Enhanced viral intrahost dynamics and increased emergence of novel viral variants has been reported in chronically norovirus-infected immunocompromised subjects (Bull et al., 2012). Also, chronic norovirus infections in immunosuppressed patients showed increased virus diversity when compared to those of acute nature (Bok and Green, 2012). The emergence of mutations that favor virus entry and persistence has also been reported among immunocompromised patients infected with human parainfluenza virus 3 (Greninger et al., 2021).

Careful and continuous monitoring of SARS-CoV-2 infecting chronically immunosuppressed patients is pivotal to successful genomic surveillance efforts currently conducted worldwide, and targeting those subjects for early treatment and halting their virus replication is *sine qua non* to the containment of the COVID-19 pandemic.

## Data availability statement

The datasets presented in this study can be found in online repositories. The names of the repository/repositories and accession number(s) can be found in the article/supplementary material.

## Ethics statement

The studies involving human participants were reviewed and approved by the Brazilian National Ethics Committee (CONEP). Written informed consent for participation was not required for this study in accordance with the national legislation and the institutional requirements.

## Author contributions

LG, JS, and MS: conceptualization and writing—original draft preparation. LG, JS, and BA: methodology and formal analysis. CC, JA, JV, and MS: resources and funding acquisition. LG, JS, MG, and BA: data curation. LG, JS, MG, BA, CC, JA, JV, and MS: writing—review and editing. All authors have read and agreed to the published version of the manuscript.

## Footnotes

1
https://artic.network/ncov-2019
